# Rate control in septic shock–associated atrial fibrillation: flow, not heart rate alone, should define haemodynamic success

**DOI:** 10.1186/s13054-026-06338-6

**Published:** 2026-09-16

**Authors:** Athanasios Chalkias

**Affiliations:** 1 Institute for Translational Medicine and Therapeutics, University of Pennsylvania Perelman School of Medicine, Philadelphia, PA 19104-5158 USA; 2 Outcomes Research Consortium®, Houston, TX 77030 USA; 3 Department of Critical Care Medicine, Tzaneio General Hospital, Piraeus, 18536 Greece

I read with considerable interest the European Delphi consensus by Guarracino et al. on the use of landiolol in septic shock complicated by new-onset atrial fibrillation (AF) [1]. The authors should be commended for placing haemodynamic assessment at the centre of therapeutic decision-making and, particularly, for emphasising cardiac output (CO), stroke volume (SV), ventricular function, preload responsiveness, and indices of tissue perfusion during heart rate (HR) control. Their recommendation that an initial HR target below 110 beats min^− 1^ should be individualised according to haemodynamic tolerance provides an important physiology-oriented framework.

I suggest that this framework can be extended one step further: from identifying what should be monitored to defining what constitutes haemodynamic success after HR reduction. The therapeutic objective of rate control should not be HR reduction per se, but preservation or improvement of effective forward flow and tissue perfusion. A decrease in HR does not have a uniform haemodynamic meaning. Its effect on CO depends critically on whether SV can increase sufficiently to compensate for the loss of chronotropic contribution to flow. This distinction is particularly relevant in septic shock, where tachycardia may predominantly impair ventricular filling in one patient yet remain an important component of haemodynamic compensation in another.

Because CO is determined by the product of HR and SV, the haemodynamic response to rate control can be described by comparing two states: *state 1*, before HR reduction, and *state 2*, after HR has been reduced and the haemodynamic response has been established. Thus, HR_1_, SV_1_, and CO_1_ represent the pre-treatment values, whereas HR_2_, SV_2_, and CO_2_ represent the corresponding values after rate control. The proportional change in CO is therefore determined by the simultaneous proportional changes in HR and SV:

$$\:\frac{C{O}_{2}}{\mathrm{C}{O}_{1}}=\frac{{HR}_{2}}{{HR}_{1}}\times\:\:\frac{{SV}_{2}}{{SV}_{1}}$$

This equation makes the central physiological point explicit. When HR decreases, HR_2_/HR_1_ becomes < 1. CO can therefore remain unchanged or increase only if the accompanying increase in SV is sufficiently large to offset the reduction in rate. Accordingly, preservation or increase of CO requires:

$$\:\frac{\mathrm{S}{V}_{2}\:}{\mathrm{S}{V}_{1}}\ge\:\:\frac{{HR}_{1}}{{HR}_{2}}$$

In other words, the required increase in SV is not numerically identical to the percentage reduction in HR. Because HR and SV interact multiplicatively to determine CO, progressively larger reductions in HR require disproportionately greater SV recruitment. For example, reducing HR from 120 to 96 beats min^− 1^ represents a 20% reduction. To maintain the same CO:

$$\mathrm{HR1/HR2} = {120}/{96} = 1.25$$

and therefore:

$${\mathrm{SV2}}/{\mathrm{SV1}}\, {\text{must be at least}}\,1.25.$$

Thus, SV must increase by 25%, rather than 20%, merely to preserve flow.

The same relationship can be translated directly into a clinically intuitive expression for the minimum SV increase required for any given percentage reduction in HR:

$$\:\mathrm{S}{V}_{required\:increase}\left(\mathrm{\%}\right)=\:\frac{{HR}_{reduction}\left(\%\right)}{{100-HR}_{reduction}\left(\%\right)}\times\:100$$

Thus, reductions in HR of 10%, 20%, 25%, and 30% require increases in SV of approximately 11%, 25%, 33%, and 43%, respectively, simply to maintain the same CO. For small changes, the required percentage increase in SV approximately equals the percentage reduction in HR; for larger HR reductions, however, the exact relation should be used because the required SV recruitment increases non-linearly. These relationships describe averaged haemodynamic states; in AF, however, neither state should be represented by an isolated cardiac cycle. Irregular RR intervals produce marked beat-to-beat variation in diastolic filling and SV, and the mechanical response of a given beat is influenced by preceding cycle lengths. HR_1_, SV_1_, and CO_1_ and HR_2_, SV_2_, and CO_2_ should therefore denote appropriately standardised multi-beat or time-averaged values before and after rate control. This averaging is integral to physiological interpretation, rather than merely a measurement convenience.

This simple relationship has important clinical implications. A numerically successful reduction in HR may still represent haemodynamic failure if SV recruitment is inadequate and CO consequently falls. I therefore propose that the response to an ultra-short-acting β_1_-blocker may usefully be conceptualised as a dynamic haemodynamic challenge, rather than solely as attainment of an absolute HR threshold. The response also has an important temporal dimension. The immediate haemodynamic effect of slowing HR may not represent the response that emerges after a modest period of adaptation. Even after the achieved HR has stabilised, time-dependent changes in myocardial wall stress, lusitropy, ventricular filling, ventricular interaction, and venous-return conditions may continue to modify SV and CO. Accordingly, rate control should be evaluated as a haemodynamic trajectory, using serial multi-beat or time-averaged measurements at predefined early and later intervals rather than relying on a single immediate post-titration value.

For physiological and research applications, the adequacy of SV recruitment relative to the imposed reduction in HR can be expressed as a dimensionless logarithmic stroke-volume compensation ratio:

$$\:\mathrm{S}\mathrm{V}\mathrm{C}\mathrm{R}_{ln}=\:\frac{\mathrm{l}\mathrm{n}(\mathrm{S}{V}_{2}\:/\:\mathrm{S}{V}_{1})}{\mathrm{l}\mathrm{n}(H{R}_{1}\:/\:H{R}_{2})}$$

For a true HR reduction, SVCRₗₙ = 1 denotes exact compensation: the proportional increase in SV is precisely sufficient to offset the proportional reduction in HR, and CO is preserved. SVCRₗₙ >1 indicates that SV recruitment exceeds that required to compensate for HR reduction and therefore that CO increases, whereas SVCRₗₙ <1 identifies incomplete compensation and a fall in CO.

For clinical practice, the same concept can be expressed more intuitively without logarithms:

$$\:\mathrm{S}\mathrm{V}\mathrm{C}\mathrm{R}=\:\frac{(\mathrm{S}{V}_{2}\:/\:\mathrm{S}{V}_{1})-1}{(H{R}_{1}\:/\:H{R}_{2})-\:1}$$

Here, the numerator represents the fractional increase in SV actually achieved, whereas the denominator represents the exact fractional increase in SV required to compensate for the observed reduction in HR. Accordingly, SVCR = 1 denotes exactly sufficient SV recruitment to preserve CO, SVCR > 1 indicates more-than-compensatory SV recruitment, and SVCR < 1 identifies inadequate compensation.

The clinical question can therefore be reduced to a simple physiological test: after HR has been lowered, did the circulation recruit enough SV to compensate for the reduction in rate? If not, an apparently successful rate-control intervention may in fact represent haemodynamic failure.

These formulations are not proposed as validated clinical endpoints. Rather, they are physiological descriptors intended to make explicit the haemodynamic question already implicit in rate-control therapy. When CO is measured directly, preservation or increase of CO remains the most immediate criterion of haemodynamic success. The compensation ratios may nevertheless provide mechanistic information by quantifying the coupling between chronotropic reduction and SV recruitment and by identifying circumstances in which apparently adequate rate control is achieved at the expense of forward flow.

The direction of this response is likely to be strongly phenotype dependent. At high ventricular rates, shortening of diastole, irregular cycle length, loss of coordinated atrial contribution, impaired ventricular filling, increased myocardial oxygen demand, and adverse ventricular–arterial interaction may constrain SV. In this phenotype, modest HR reduction may prolong effective filling sufficiently to recruit SV, potentially preserving or even increasing CO despite the lower rate.

Rate reduction also has a myocardial energetic dimension. Tachycardia increases myocardial oxygen consumption while shortening diastole, the predominant period of coronary perfusion; slowing HR may therefore reduce oxygen demand and increase the time available for myocardial oxygen supply [2]. However, the net energetic effect depends on both demand and delivery. Adequate myocardial oxygen supply requires preserved diastolic coronary perfusion pressure and arterial oxygen content, while wall stress and contractile state remain important determinants of demand. If HR reduction lowers CO or diastolic arterial pressure, or if ventricular filling pressures and wall stress remain excessive, myocardial oxygen supply-demand balance may fail to improve despite a lower HR. Myocardial energetics should therefore be considered alongside systemic flow and tissue perfusion when judging haemodynamic success.

Conversely, when SV is constrained by impaired contractility, inadequate preload, right-ventricular (RV) dysfunction, or vasoplegia associated with limited stressed-volume and venous-return reserve, the higher ventricular rate may contribute materially to maintaining systemic flow. HR reduction in such patients may expose limited SV reserve and precipitate a fall in CO. The clinically relevant distinction is therefore not merely whether tachycardia is present, but whether it is predominantly maladaptive or compensatory within the prevailing haemodynamic phenotype.

This distinction may also help interpret the broader experience with β-blockade in sepsis. J-Land 3 S demonstrated effective HR control with landiolol in patients with sepsis-related tachyarrhythmia [3]. Although not AF-specific, Landi-SEP did not support stringent HR lowering below 95 beats/min as a uniform strategy in an unselected septic-shock population, further highlighting the importance of identifying haemodynamic phenotypes likely to benefit from HR control [4]. Taken together, these observations suggest that the relevant treatment variable may not be HR alone, but the capacity of the individual circulation to convert HR reduction into effective SV recruitment and preserved systemic flow.

The venous circulation and right heart are central to this response. At haemodynamic steady state, CO must equal venous return. Consequently, an increase in SV after HR reduction can occur only if the venous-return circulation can sustain adequate filling at the lower rate and the RV–pulmonary circulation can transmit that flow effectively. The pressure gradient driving venous return is determined conceptually by the difference between mean systemic filling pressure and right atrial pressure, together with resistance to venous return. A patient with depleted effective stressed volume or limited venous-return reserve may therefore be unable to increase SV sufficiently when HR falls. Equally importantly, elevation of right atrial pressure caused by RV dysfunction reduces the pressure gradient for venous return and may impose a ceiling on forward flow.

This right-heart dependence deserves particular attention in septic shock. AF, pulmonary vascular dysfunction, mechanical ventilation, positive end-expiratory pressure, and septic myocardial dysfunction may converge to impair RV filling and ejection. A slower ventricular rate may improve coronary perfusion and diastolic filling in some patients, yet it cannot guarantee greater RV SV when RV afterload is excessive or contractile reserve is exhausted [5]. HR therefore cannot be interpreted independently of the venous-return–RV–pulmonary circulation–left ventricular continuum.

I therefore suggest that successful rate control in septic shock-associated AF should be defined physiologically as HR reduction accompanied by preserved or increased CO, appropriate compensatory SV recruitment, maintained arterial pressure without escalation of vasoactive support, and stable or improving tissue perfusion.

Conversely, falling CO, SVCR < 1, absent or inadequate SV recruitment, increasing vasopressor requirements, rising venous pressures or worsening venous congestion, prolonged capillary refill, declining central venous oxygen saturation, or deterioration in other perfusion indices should signal that further HR reduction may be haemodynamically inappropriate (Fig. 1). Importantly, preservation of arterial pressure alone cannot establish haemodynamic success, because vascular tone and vasoactive therapy may maintain pressure despite deterioration in forward flow.

**Fig. 1:**
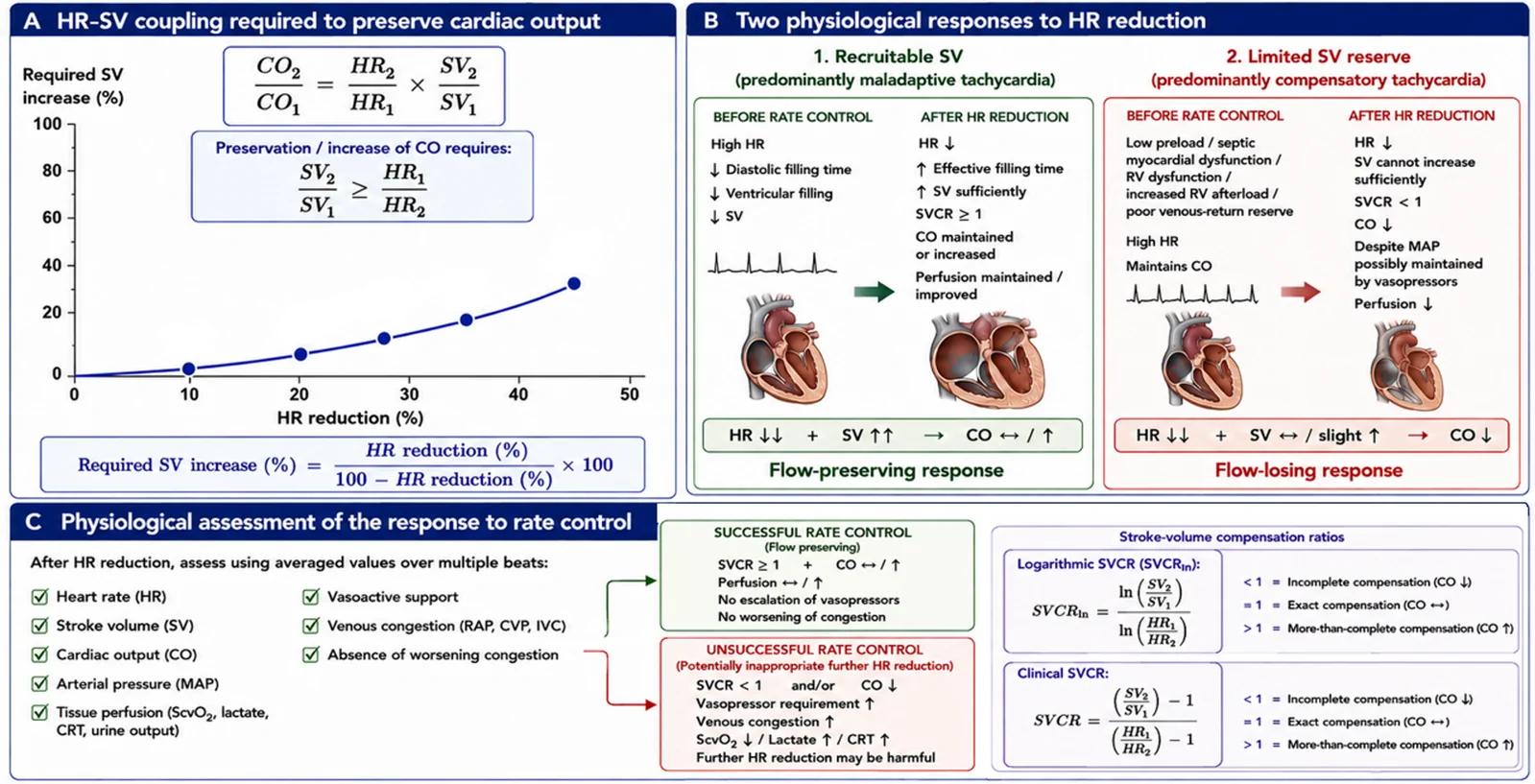
Heart rate–stroke-volume coupling and physiological assessment of rate control in septic shock-associated atrial fibrillation. **(A)** Relationship between HR reduction and the compensatory increase in SV required to preserve CO. The proportional change in CO after rate control is determined by the coupled changes in HR and SV: CO_2_/CO_1_ = (HR_2_/HR_1_) × (SV_2_/SV_1_), while preservation or increase of CO therefore requires SV_2_/SV_1_ ≥ HR_1_/HR_2_. The curve illustrates the non-linear increase in SV recruitment required as the magnitude of HR reduction increases; plotted points are illustrative. **(B)** Conceptual haemodynamic phenotypes following HR reduction. In patients with recruitable SV and predominantly maladaptive tachycardia, prolongation of effective filling time may permit sufficient SV recruitment, resulting in an SV compensation ratio (SVCR) ≥ 1, preserved or increased CO, and maintained or improved tissue perfusion. Conversely, when tachycardia is predominantly compensatory, HR reduction may not be accompanied by sufficient SV recruitment (SVCR < 1), resulting in reduced CO despite potentially preserved mean arterial pressure through vasoactive support. **(C)** Proposed physiological assessment of the response to rate control using HR, SV, CO, arterial pressure, vasoactive requirements, venous congestion, and tissue-perfusion indices, with haemodynamic variables averaged over multiple beats because of the beat-to-beat variability inherent to atrial fibrillation and, where feasible, repeated at predefined early and later intervals after HR stabilisation to capture temporal adaptation. Successful rate control is characterised by adequate SV recruitment, preserved or increased CO, stable or improving perfusion, no escalation of vasopressor support, and no worsening congestion. The logarithmic stroke-volume compensation ratio (SVCRₗₙ) and the clinically simpler SVCR quantify the adequacy of SV recruitment relative to the imposed HR reduction. Values < 1 indicate incomplete compensation with a tendency toward reduced CO, a value of 1 indicates exact compensation, and values > 1 indicate more-than-complete compensation with preserved or increased CO. AF, atrial fibrillation; CO, cardiac output; CO_1_, cardiac output before rate control; CO_2_, cardiac output after rate control; CRT, capillary refill time; CVP, central venous pressure; HR, heart rate; HR_1_, heart rate before rate control; HR_2_, heart rate after rate control; IVC, inferior vena cava; MAP, mean arterial pressure; RAP, right atrial pressure; RV, right ventricle; ScvO_2_, central venous oxygen saturation; SV, stroke volume; SV_1_, stroke volume before rate control; SV_2_, stroke volume after rate control; SVCR, stroke-volume compensation ratio

Future research should therefore characterise the response to rate control with serial, standardised multi-beat or time-averaged measurements at predefined intervals after HR reduction, integrating HR–SV–CO coupling with biventricular performance, venous-return reserve and congestion, myocardial oxygen supply-demand balance, vasoactive requirements, and tissue perfusion. Such an approach may permit identification of phenotypes in which slowing HR improves cardiovascular performance and, equally importantly, those in which tachycardia continues to contribute to haemodynamic compensation.

## Data Availability

No datasets were generated or analysed during the current study.
